# Risk Factors and Incidence of Colorectal Cancer According to Major Molecular Subtypes

**DOI:** 10.1093/jncics/pkaa089

**Published:** 2020-10-07

**Authors:** Liang Wang, Xiaosheng He, Tomotaka Ugai, Koichiro Haruki, Chun-Han Lo, Dong Hang, Naohiko Akimoto, Kenji Fujiyoshi, Molin Wang, Charles S Fuchs, Jeffrey A Meyerhardt, Xuehong Zhang, Kana Wu, Andrew T Chan, Edward L Giovannucci, Shuji Ogino, Mingyang Song

**Affiliations:** 1 Center of Gastrointestinal Surgery, the First Affiliated Hospital, Sun Yat-Sen University, Guangzhou, P.R. China; 2 Department of Epidemiology, Harvard T. H. Chan School of Public Health, Boston, MA, USA; 3 Department of Colorectal Surgery, the Six Affiliated Hospital, Sun Yat-sen University, Guangzhou, P.R. China; 4 Department of Pathology, Brigham and Women’s Hospital and Harvard Medical School, Program in MPE Molecular Pathological Epidemiology, Boston, MA, USA; 5 Division of Gastroenterology, Massachusetts General Hospital and Harvard Medical School, Boston, MA, USA; 6 Department of Epidemiology and Biostatistics, Jiangsu Key Lab of Cancer Biomarkers, Prevention and Treatment, Collaborative Innovation Center for Cancer Personalized Medicine, School of Public Health, Nanjing Medical University, Nanjing, P.R. China; 7 Department of Biostatistics, Harvard T. H. Chan School of Public Health, Boston, MA, USA; 8 Department of Medicine, Yale Cancer Center, New Haven, CT, USA; 9 Department of Medicine, Yale School of Medicine, New Haven, CT, USA; 10 Department of Medicine, Smilow Cancer Hospital, New Haven, CT, USA; 11 Department of Medical Oncology, Dana-Farber Cancer Institute and Harvard Medical School, Boston, MA, USA; 12 Channing Division of Network Medicine, Department of Medicine, Brigham and Women’s Hospital and Harvard Medical School, Boston, MA, USA; 13 Department of Nutrition, Harvard T. H. Chan School of Public Health, Boston, MA, USA; 14 Broad Institute of MIT and Harvard, Cambridge, MA, USA; 15 Clinical and Translational Epidemiology Unit, Massachusetts General Hospital and Harvard Medical School, Boston, MA, USA; 16 Department of Immunology and Infectious Diseases, Harvard T. H. Chan School of Public Health, Boston, MA, USA; 17 Cancer Immunology and Cancer Epidemiology Programs, Dana-Farber Harvard Cancer Center, Boston, MA, USA

## Abstract

**Background:**

Colorectal cancer (CRC) is a heterogeneous disease that can develop via 3 major pathways: conventional, serrated, and alternate. We aimed to examine whether the risk factor profiles differ according to pathway-related molecular subtypes.

**Methods:**

We examined the association of 24 risk factors with 4 CRC molecular subtypes based on a combinatorial status of microsatellite instability (MSI), CpG island methylator phenotype (CIMP), and *BRAF* and *KRAS* mutations by collecting data from 2 large US cohorts. We used inverse probability weighted duplication-method Cox proportional hazards regression to evaluate differential associations across subtypes.

**Results:**

We documented 1175 CRC patients with molecular subtype data: subtype 1 (n = 498; conventional pathway; non-MSI-high, CIMP-low or negative, *BRAF*-wild-type, *KRAS*-wild-type), subtype 2 (n = 138; serrated pathway; any MSI status, CIMP-high, *BRAF*-mutated, *KRAS*-wild-type), subtype 3 (n = 367; alternate pathway; non-MSI-high, CIMP-low or negative, *BRAF*-wild-type, *KRAS*-mutated), and subtype 4 (n = 172; other marker combinations). Statistically significant heterogeneity in associations with CRC subtypes was found for age, sex, and smoking, with a higher hazard ratio (HR) observed for the subtype 2 (HR per 10 years of age = 2.64, 95% CI = 2.13 to 3.26; HR for female = 2.65, 95% CI = 1.60 to 4.39; HR per 20-pack-year of smoking = 1.29, 95% CI = 1.14 to 1.45) than other CRC subtypes (all *P*_heterogeneity_ < .005). A stronger association was found for adiposity measures with subtype 1 CRC in men and subtype 3 CRC in women and for several dietary factors with subtype 1 CRC, although these differences did not achieve statistical significance at α  level of .005.

**Conclusions:**

Risk factor profiles may differ for CRC arising from different molecular pathways.

Colorectal cancer (CRC) is a heterogeneous disease that can develop via 3 major pathways: conventional, serrated, and alternate (1,2). These pathways are associated with certain combinations of major molecular features of CRC, including microsatellite instability (MSI), the CpG island methylator phenotype (CIMP), and somatic mutations in *BRAF* and *KRAS*. The “conventional” adenoma-carcinoma pathway is characterized by non-MSI-high and CIMP-low or negative, and no mutations in *BRAF* or *KRAS*; the “serrated” pathway by frequent mutation in *BRAF* and CIMP-high; and the “alternate” pathway by *KRAS* mutation and non-MSI-high or CIMP-low or negative (1–3). Prognostic studies have shown that patients with CRC arising from these distinct pathways have considerably different survival (3,4).

Similarly, increasing evidence indicates the etiologic heterogeneity of CRC. Recently, we performed a comprehensive analysis of the risk factor profiles for serrated polyps and conventional adenomas, the 2 major precursors of CRC (5). We identified distinct risk factor profiles for the 2 CRC precursors (5). Moreover, the influence of these risk factors on CRC risk has been shown to vary according to tumor molecular characteristics (6). However, most of the existing studies have focused on individual molecular characteristics of CRC (7–9). Given that the CRC pathways are typically characterized by multiple molecular features that commonly co-occur in each specific tumor, a comprehensive assessment of risk factors in relation to CRC subtypes defined by a combination of multiple molecular features represents the next critical step to better understand the etiology of CRC.

Therefore, leveraging data of 2 large prospective cohort studies with 3063 incident CRC patients in total and a molecular pathological epidemiology database of 1175 CRC patients, we characterized the risk factor profiles of 4 CRC subtypes related to the conventional, serrated, alternate, and other pathways. We aimed to examine whether 24 established CRC risk factors differentially associated with incidence of CRC subtypes.

## Methods

### Study Population

The Nurses’ Health Study (NHS) enrolled 121 700 US registered female nurses aged 30-55 years in 1976. The Health Professionals Follow-up Study (HPFS) enrolled 51 529 US male health professionals aged 40-75 years in 1986. Details about the 2 cohorts have been described elsewhere (10,11). Briefly, participants were mailed a questionnaire inquiring about their medical history and lifestyle factors at baseline and every 2 years thereafter. Dietary data were collected and updated every 4 years using the validated semi-quantitative food frequency questionnaires (FFQs) beginning in 1980 in the NHS and 1986 in the HPFS. In the present analysis, we used 1980 for the NHS and 1986 for the HPFS as baseline.

At baseline, we excluded participants with a history of inflammatory bowel disease and cancer (except for nonmelanoma skin cancer), missing lifestyle data, and those with missing FFQs or a high number of blank items on their FFQs (>70), with implausibly high or low caloric intakes (ie, <800 or >4200 kcal/d for men; <600 or >3500 kcal/d for women). This study was approved by the institutional review board at the Brigham and Women’s Hospital and the Harvard T. H. Chan School of Public Health and those of participating registries as required.

### Ascertainment of CRC patients

On each biennial follow-up questionnaire, participants were asked whether they were diagnosed with CRC during the previous 2 years. For participants who reported a diagnosis of CRC, we asked for their permission to acquire medical records and pathologic reports. Study physicians, blinded to exposure data, reviewed all medical records to confirm CRC diagnosis and to record the disease stage, histologic findings, and tumor location.

### Tumor Molecular Marker Assessment

We collected formalin-fixed paraffin-embedded tissue blocks from the hospitals throughout the United States where participants with CRC had undergone surgery. Details of tumor molecular assays have been described elsewhere (12–14). Briefly, we performed real-time polymerase chain reaction (PCR) and pyrosequencing targeted for *KRAS* codons 12, 13, 61, and 147 (13) and *BRAF* codon 600 (12). Status was determined using 10 microsatellite markers (D17S250, D18S55, D18S56, D18S67, D18S487, D2S123, D5S346, BAT25, BAT26, and BAT40), and tumors were classified as MSI-high if 30% or more of the markers demonstrated instability (12). We quantified DNA methylation using bisulfite modification and the MethyLight assay (14) on 8 CpG island methylator phenotype (CIMP)-specific promoters [*MLH1*, *NEUROG1*, *RUNX3*, *CACNA1G*, *CDKN2A* (p16), *CRABP1*, *IGF2*, and *SOCS1*] and classified tumors as CIMP-high if 6 or more promoters were methylated and as CIMP-low or negative if 0 to 5 promoters were methylated (12).

### Molecular Subtype Classifications

We used the molecular markers to define 4 subtypes of CRC based on the previously proposed classifications (1,2): subtype 1 (conventional pathway) is defined as non-MSI-high, CIMP-low or negative, *BRAF*-wild-type, and *KRAS*-wild-type CRC; subtype 2 (serrated pathway) as any MSI status, CIMP-high, *BRAF*-mutated, and *KRAS*-wild-type CRC; subtype 3 (alternate pathway) as non-MSI-high, CIMP-low or negative, *BRAF*-wild-type, and *KRAS*-mutated CRC; and subtype 4 (the other pathway) as CRC with the other combinations of the 4 markers.

### Risk Factor Assessment

On each biennial questionnaire, we assessed age, family history of CRC in a first-degree relative, smoking history, body mass index (BMI), BMI at age 18 years for females and age 21 years for males, leisure-time physical activity, aspirin use, use of endoscopic exams, and alcohol consumption. Details of assessment of these risk factors are described in the Supplementary Methods (available online). Using the semi-quantitative FFQ data, we assessed several dietary factors that have been associated with CRC, including total dietary fiber, cereal fiber, whole grains, total red meat, processed red meat, unprocessed red meat, folate, calcium, marine n-3 fatty acid intake (including eicosapentaenoic acid, docosahexaenoic acid, and docosapentaenoic acid), and vitamin D (15). Supplement use was included in the calculation of total nutrient intake, which was further adjusted for total caloric intake by the residual method (16). Furthermore, to examine the overall dietary patterns, we calculated 2 empirically derived dietary indices, including empirical dietary inflammatory pattern (EDIP) and empirical dietary index for hyperinsulinemia, which have shown robust associations with inflammatory and insulin biomarkers, respectively (17,18).

### Statistical Analysis

Participants were followed until the diagnosis of CRC, death, or the end of the follow-up (June 1, 2014), whichever occurred first. To capture long-term exposure, we calculated the cumulative average of risk factors from preceding questionnaires up to the current cycle. Leveraging covariate data of 3063 incident CRC patients with or without tumor tissue, we used inverse probability weighting (IPW) method to adjust for selection bias because of tissue availability (19,20). Subtype-stratified multivariable IPW-adjusted Cox proportional hazards regression models with a duplication method (21) were used to calculate the hazard ratio (HR) and its 95% confidence interval (CI) for each molecular subtype of CRC in relation to risk factors and to assess heterogeneity of differential associations with CRC subtypes. We used age as the time scale and stratified by sex and calendar time in the Cox models. All models were adjusted for race and the nondietary risk factors. Details of statistical analysis are described in the Supplementary Methods (available online). We calculated the hazard ratios per certain increment for continuous variables based on the literature and to reflect the distribution of the studied exposure in the US population. Given the known sex difference in the effect of adiposity on CRC, we examined sex-specific associations for BMI and waist circumference. A test of heterogeneity was conducted using a likelihood ratio test that compared the model that allowed for different associations of risk factors according to molecular subtypes with a model that assumed a common effect (21). We calculated 2 sets of *P* for heterogeneity, including a global test across all CRC subtypes and a pairwise test between subtype 1 and each of the other subtypes.

To control for multiple testing, we used a stringent α level of .005 [as recommended by Benjamin et al. (22)] for our primary hypothesis testing of the heterogeneity in the associations between risk factors and CRC across molecular subtypes. All other analyses, including evaluation of individual hazard ratios, represented secondary analyses. All the analyses were performed using SAS 9.4 (SAS Institute, Cary, NC). All tests of statistical significance were 2-sided.

## Results

### Cohorts Characteristics and Main Findings

**Figure 1.**
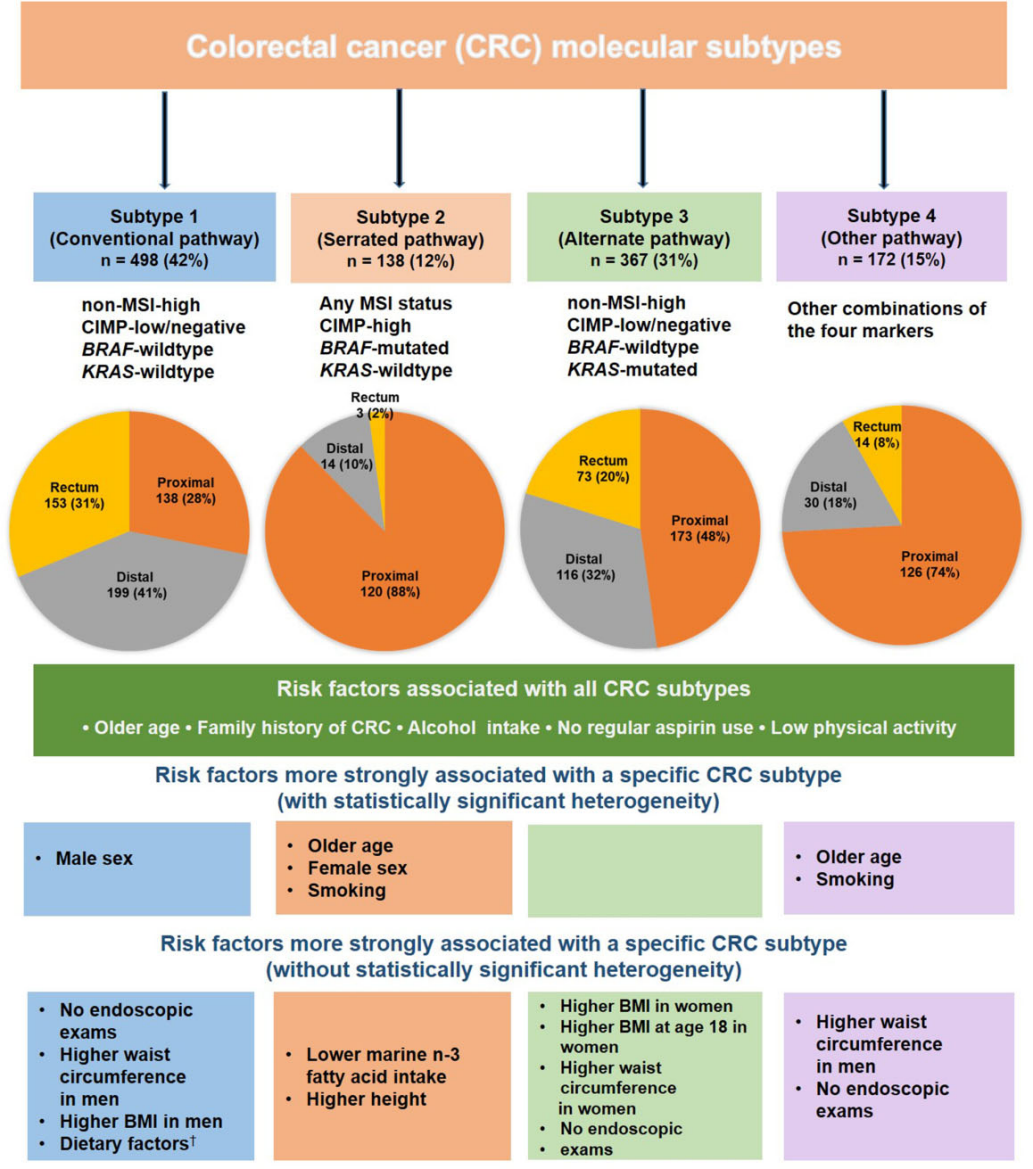
Subsite distribution and risk factor profiles of colorectal cancer (CRC) according to molecular subtypes. The bottom panels demonstrate risk factors that had a particularly stronger association with a specific subtype of CRC compared with other subtypes. For CRC subsites, tumors with missing subsite information are not included in the pie charts. ^†^Dietary factors include less whole grains intake, less cereal fiber intake, less vitamin D intake, less folate intake, less calcium intake, higher total red meat intake, higher processed red meat intake, and higher unprocessed red meat intake. BMI = body mass index; CIMP = CpG island methylator phenotype; CRC = colorectal cancer; MSI = microsatellite instability.

Among 131 331 participants in the NHS and HPFS cohorts followed for a median of 30 years, we documented 3063 incident CRC patients including 1175 CRC patients with available data on molecular subtypes, including 498 subtype 1 patients, 138 subtype 2 patients, 367 subtype 3 patients, and 172 subtype 4 patients . Compared with other subtypes, patients with subtype 2 were more likely to be older, females, and smokers and had a higher fraction of proximal colon cancer (Table 1). The risk factor characteristics did not appear to differ between patients with and without tissue specimens (Supplementary Table 1, available online). The main findings are summarized in Figure 1 and Tables 2-4. Briefly, statistically significant heterogeneity in associations with CRC subtypes was found for age, sex, and smoking, with a higher hazard ratio observed for the subtype 2 than other CRC subtypes (all *P*_heterogeneity_ < .005). A stronger association was found for adiposity measures with subtype 1 CRC in men and subtype 3 CRC in women and for several dietary factors with subtype 1 CRC, although these differences did not achieve statistical significance at α  level of .005. The Cox regression analyses without IPW yielded similar results to the IPW-adjusted model.

**Table 1.** Basic characteristics of study participants in the NHS and HPFS cohorts^a^

Characteristics	Participants without CRC (n = 120 800)	Participants with CRC				
		All CRC (n = 3063)	Subtype 1^b^ (Conventional pathway) (n = 498)	Subtype 2^b^ (Serrated pathway) (n = 138)	Subtype 3^b^ (Alternate pathway) (n = 367)	Subtype 4^b^ (Other pathway) (n = 172)
Age, mean (SD), y	62.0 (11.7)	67.5 (9.7)	65.8 (9.2)	70.1 (7.6)	67.4 (8.8)	68.0 (8.8)
White, %	96.5	97.0	95.3	95.8	96.6	96.8
Female, %	71.4	58.2	55.0	78.3	57.2	64.0
Family history of CRC, %	15.0	22.0	23.7	25.8	22.6	36.1
Regular aspirin use, %^c^	41.8	35.6	38.2	49.9	38.1	44.8
Height,mean (SD), cm	168 (9)	168 (9)	170 (9)	171 (9)	170 (10)	170 (10)
BMI, mean (SD), kg/m^2^	26.0 (4.8)	26.3 (5.0)	26.7 (4.4)	26.3 (5.3)	26.2 (4.7)	26.2 (4.0)
BMI at age 18/21 years, mean(SD), kg/m^2 f^	21.7 (3.5)	21.8 (3.7)	22.1 (3.6)	21.4 (3.7)	22.0 (3.7)	22.1 (3.8)
Waist circumference, mean (SD), cm	86.3 (13.3)	87.7 (13.2)	90.88 (13.0)	89.95 (14.7)	89.47 (13.8)	90.3 (13.8)
Pack-years of smoking, mean (SD)	12.9 (18.9)	15.0 (21.1)	16.0 (21.6)	21.9 (24.3)	16.2 (20.7)	19.4 (22.7)
Pack-years of smoking before age 30 years, mean (SD)	4.2 (5.9)	4.4 (6.0)	4.6 (6.3)	5.7 (6.0)	4.9 (5.8)	5.5 (6.8)
Physical activity, mean (SD), METS-hours/wk^d^	20.7 (20.8)	19.5 (18.4)	18.3 (17.2)	19.0 (15.6)	19.5 (19.7)	16.7 (15.9)
Alcohol intake, mean (SD), g/day	7.5 (11.0)	8.3 (12.2)	9.4 (13.8)	9.4 (13.0)	9.6 (14.5)	9.5 (12.5)
Total folate intake, mean (SD), µg/d	463 (231)	434 (220)	449 (207)	460 (180)	463 (191)	460 (194)
Calcium intake, mean (SD), mg/d	939 (363)	883 (355)	893 (352)	935 (310)	950 (372)	934 (342)
Vitamin D intake, mean (SD), IU/d	383 (238)	355 (234)	364 (225)	368 (206)	371 (220)	382 (219)
Marine n-3 fatty acid intake, mean (SD), g/d	0.22 (0.19)	0.21 (0.18)	0.24 (0.19)	0.19 (0.10)	0.24 (0.24)	0.23 (0.19)
Total red meat intake, mean (SD), serving/wk	6.5 (3.9)	6.7 (4.0)	6.6 (3.5)	6.4 (3.5)	6.0 (3.5)	6.2 (3.5)
Processed red meat intake, mean (SD), serving/wk	2.1 (2.1)	2.3 (2.2)	2.3 (2.1)	2.1 (1.8)	1.9 (1.7)	2.1 (2.0)
Unprocessed red meat intake, mean (SD), serving/wk	4.1 (2.6)	4.2 (2.5)	4.0 (2.2)	4.0 (2.2)	3.8 (2.4)	3.8 (2.3)
Total fiber intake, mean (SD), g/d	18.0 (6.0)	17.8 (6.0)	18.9 (5.8)	19.1 (5.9)	19.2 (6.1)	19.2 (6.6)
Whole grain intake, mean (SD), g/d	20.4 (14.8)	19.4 (14.4)	19.1 (13.0)	21.8 (17.0)	21.4 (13.1)	20.6 (16.4)
Cereal fiber intake, mean (SD), g/d	4.77 (2.72)	4.62 (2.50)	4.91 (2.45)	5.38 (2.52)	5.22 (2.57)	5.36 (3.49)
Empirical dietary inflammatory pattern score, mean (SD)^e^	−0.02 (0.33)	0.01 (0.35)	0 (0.33)	0 (0.33)	−0.04 (0.33)	0.01 (0.36)
Empirical dietary index for hyperinsulinemia, mean (SD)^e^	0.33 (0.29)	0.35 (0.30)	0.36 (0.25)	0.37 (0.26)	0.33 (0.25)	0.37 (0.27)
Tumor location, No. (%)						
Proximal colon	—	1281 (41.8)	138 (27.7)	120 (87.0)	173 (47.1)	126 (73.3)
Distal colon	—	879 (28.7)	199 (40.0)	14 (10.1)	116 (31.6)	30 (17.4)
Rectum	—	653 (21.3)	153 (30.7)	3 (2.2)	73 (19.9)	14 (8.1)
Missing	—	250 (8.2)	8 (1.6)	1 (0.7)	5 (1.4)	2 (1.2)
Tumor TNM Stage, No. (%)^g^						
Stage I	—	620 (20.2)	130 (26.1)	23 (16.7)	82 (22.3)	39 (22.7)
Stage II	—	669 (21.8)	127 (25.5)	63 (45.7)	93 (25.3)	60 (34.9)
Stage III	—	652 (21.3)	142 (28.5)	33 (23.9)	104 (28.3)	33 (19.2)
Stage IV	—	508 (16.6)	45 (9.0)	14 (10.1)	62 (16.9)	29 (16.9)
Missing	—	614 (20.0)	54 (10.8)	5 (3.6)	26 (7.1)	11 (6.4)

a For participants without CRC, the data are based on the average of information throughout the follow-up, whereas for CRC patients the data at diagnosis are presented. All variables are adjusted for age and sex except for age and sex themselves. BMI = body mass index; CIMP = CpG island methylator phenotype; CRC = colorectal cancer; HPFS = Health Professionals Follow-up Study; METS = metabolic equivalent task score; MSI = microsatellite instability; NHS = Nurses’ Health Study.

b Subtype 1 (conventional pathway; non-MSI-high, CIMP-lowor negative, *BRAF*-wild-type, and *KRAS*-wild-type); Subtype 2 (serrated pathway; any MSI status, CIMP-high, *BRAF*-mutated, and *KRAS*-wild-type); Subtype 3 (alternate pathway; non-MSI-high, CIMP-lowor negative, *BRAF*-wild-type, and *KRAS*-mutated); Subtype 4 (other marker combinations).

c A standard tablet contains 325 mg aspirin, and regular users were defined as those who used at least 2 standard tablets per week.

d Physical activity is calculated by the product sum of the METS of each specific recreational activity and hours spent on that activity per week. For physical activity, the follow-up started in 1986 in NHS.

e The range of empirical dietary inflammatory pattern was -7.59 to 9.86, and the range of empirical dietary index for hyperinsulinemia was -2.16 to 20.43.

f BMI at age 18/21, BMI at age 18 for female and age 21 for male.

g The abbreviation “TNM” stands for tumor (T), nodes (N), and metastases (M).

**Table 2.** Multivariable associations of demographic and clinical factors with risk of colorectal cancer according to molecular subtypes in the NHS and HPFS^a^

Risk factors	All CRC (n = 3063)	Subtype 1^b^ (Conventional pathway) (n = 498)	Subtype 2^b^ (Serrated pathway) (n = 138)	Subtype 3^b^ (Alternate pathway) (n = 367)	Subtype 4^b^ (Other pathway) (n = 172)	*P*_overall_ _heterogeneity_ ^c^
Age, per 10 y						
HR (95% CI)	2.05 (1.96 to 2.15)	1.70 (1.51 to 1.92)	2.64 (2.13 to 3.26)	1.84 (1.58 to 2.13)	2.34 (1.87 to 2.93)	—
*P*_trend_	<.001	<.001	<.001	<.001	<.001	.001
*P*_heterogeneity (compared to subtype 1)_^c^	—	—	<.001	.43	.01	—
Female sex						
HR (95% CI)	0.83 (0.73 to 0.93)	0.74 (0.59 to 0.92)	2.65 (1.60 to 4.39)	0.90 (0.68 to 1.18)	1.25 (0.84 to 1.85)	—
*P*	.002	.007	<.001	.45	.27	<.001
*P*_heterogeneity (compared to subtype 1)_^c^	—	—	<.001	.27	.02	—
Family history of colorectal cancer						
HR (95% CI)	1.43 (1.32 to 1.56)	1.54 (1.22 to 1.94)	1.64 (1.11 to 2.43)	1.21 (0.93 to 1.57)	2.05 (1.43 to 2.93)	—
*P*	<.001	<.001	.01	.17	<.001	.13
*P*_heterogeneity (compared to subtype 1)_^c^	—	—	.79	.17	.19	—
Endoscopic exams						
HR ( 95% CI)	0.54 (0.47 to 0.62)	0.48 (0.33 to 0.71)	0.96 (0.54 to 1.71)	0.67 (0.44 to 1.02)	0.67 (0.36 to 1.24)	—
*P*	<.001	<.001	.90	.06	.20	.25
*P*_heterogeneity (compared to subtype 1)_^c^	—	—	.05	.26	.38	—
Regular aspirin use						
HR (95% CI)	0.72 (0.67 to 0.78)	0.68 (0.55 to 0.83)	0.78 (0.55 to 1.11)	0.81 (0.63 to 1.03)	0.98 (0.70 to 1.39)	—
*P*	<.001	<.001	.17	.08	.93	.32
*P*_heterogeneity (compared to subtype 1)_^c^	—	—	.50	.29	.07	—

a Inverse probability weighting was applied to reduce a bias due to the availability of tumor tissue after cancer diagnosis (see Statistical Analysis subsection for details). Age- and cohort-stratified Cox proportional hazards model was used with further adjustment for race (White or nonWhite), height (continuous), family history of colorectal cancer (yes or no), history of lower gastrointestinal endoscopic exams (yes or no), body mass index (continuous), pack-years of smoking (continuous), physical activity (continuous), alcohol intake (continuous), and regular aspirin use (yes or no). CI = confidence interval; CIMP = CpG island methylator phenotype; CRC = colorectal cancer; HPFS = Health Professionals Follow-up Study; MSI = microsatellite instability; NHS = Nurses’ Health Study.

b Subtype 1 (conventional pathway; non-MSI-high, CIMP-lowor negative, *BRAF*-wild-type, and *KRAS*-wild-type); Subtype 2 (serrated pathway; any MSI status, CIMP-high, *BRAF*-mutated, and *KRAS*-wild-type); Subtype 3 (alternate pathway; non-MSI-high, CIMP-lowor negative, *BRAF*-wild-type, and *KRAS*-mutated); Subtype 4 (other marker combinations).

c Heterogeneity across the 4 subtypes was tested by a weighted likelihood ratio test.

**Table 3.** Multivariable associations of lifestyle and anthropometric factors with risk of colorectal cancer according to molecular subtypes in the NHS and HPFS^a^

Risk factors	All CRC (n = 3063)	Subtype 1^b^ (Conventional pathway) (n = 498)	Subtype 2^b^ (Serrated pathway) (n = 138)	Subtype 3^b^ (Alternate pathway) (n = 367)	Subtype 4^b^ (Other pathway) (n = 172)	*P*_overall_ _heterogeneity_ ^c^
Smoking						
Pack-years of smoking, per 20 pack-year						
HR (95% CI)	1.09 (1.05 to 1.12)	0.94 (0.84 to 1.04)	1.29 (1.14 to 1.45)	1.04 (0.95 to 1.14)	1.23 (1.05 to 1.43)	—
*P*_trend_	<.001	.24	<.001	.42	.01	<.001
*P*_heterogeneity (compared to subtype 1)_^c^	—	—	<.001	.15	.005	—
Pack-years of smoking before age 30 years, per 20 pack-year						
HR (95% CI)	1.18 (1.06 to 1.33)	0.76 (0.52 to 1.10)	1.52 (1.02 to 2.26)	1.04 (0.76 to 1.42)	1.60 (1.02 to 2.51)	—
*P*_trend_	.004	.14	.04	.81	.04	.03
*P*_heterogeneity (compared to subtype 1)_^c^	—	—	.01	.19	.01	—
Alcohol intake, per 14 g/d						
HR (95% CI)	1.11 (1.07 to 1.15)	1.09 (0.98 to 1.20)	1.14 (0.94 to 1.38)	1.22 (1.07 to 1.38)	1.24 (1.03 to 1.49)	—
*P*_trend_	<.001	.10	.19	.002	.02	.43
*P*_heterogeneity (compared to subtype 1)_^c^	—	—	.66	.16	.22	—
BMI, per 5 kg/m^2^						
Men	1282	224	30	157	62	—
HR (95% CI)	1.30 (1.20 to 1.40)	1.42 (1.21 to 1.67)	0.82 (0.42 to 1.60)	1.00 (0.79 to 1.27)	1.27 (0.91 to 1.77)	—
*P*_trend_	<.001	<.001	.55	.99	.15	.06
*P*_heterogeneity (compared to subtype 1)_^c^	—	—	.12	.02	.56	—
Women	1781	274	108	210	110	—
HR (95% CI)	1.16 (1.10 to 1.22)	1.10 (0.88 to 1.38)	1.06 (0.89 to 1.25)	1.23 (1.08 to 1.41)	1.06 (0.88 to 1.27)	—
*P*_trend_	<.001	.39	.54	.002	.55	.40
*P*_heterogeneity (compared to subtype 1)_^c^	—	—	.76	.40	.77	—
BMI at age 18/21, per 5 kg/m^2 e^						
Men	1282	224	30	157	62	—
HR (95% CI)	1.07 (1.00 to 1.14)	1.20 (1.03 to 1.41)	0.89 (0.73 to 1.09)	1.17 (0.99 to 1.39)	1.18 (0.89 to 1.56)	—
*P*_trend_	.06	.02	.27	.07	.24	.11
*P*_heterogeneity (compared to subtype 1_ ^c^	—	—	.02	.82	.90	—
Women	1781	274	108	210	110	—
HR (95% CI)	1.21 (1.12 to 1.30)	1.05 (0.77 to 1.44)	1.12 (0.89 to 1.41)	1.30 (1.18 to 1.45)	1.34 (1.15 to 1.60)	—
*P*_trend_	<.001	.75	.34	.007	.01	.50
*P*_heterogeneity (compared to subtype 1)_^c^	—	—	.76	.26	.23	—
Waist circumference, per 10 cm						
Men	1282	224	30	157	62	—
HR (95% CI)	1.30 (1.22 to 1.38)	1.34 (1.19 to 1.52)	1.00 (0.69 to 1.46)	1.05 (0.88 to 1.26)	1.37 (1.07 to 1.74)	—
*P*_trend_	<.001	<.001	.97	.57	.01	.08
*P*_heterogeneity (compared to subtype 1)_^c^	—	—	.15	.02	.90	—
Women	1781	274	108	210	110	—
HR (95% CI)	1.07 (1.02 to 1.12)	0.97 (0.80 to 1.18)	0.99 (0.86 to 1.14)	1.08 (0.97 to 1.20)	0.96 (0.79 to 1.18)	—
*P*_trend_	.007	.77	.89	.16	.71	.61
*P*_heterogeneity (compared to subtype 1)_^c^	—	—	.88	.35	.95	—
Height, per 10 cm						
HR (95% CI)	1.17 (1.10 to 1.23)	1.03 (0.89 to 1.18)	1.52 (1.21 to 1.90)	1.22 (1.01 to 1.47)	1.21 (0.91 to 1.61)	—
*P*_trend_	<.001	.71	<.001	.03	.19	.04
*P*_heterogeneity (compared to subtype 1)_^c^	—	—	.004	.14	.31	—
Physical activity, per 7.5 METS-hr/wk^d^						
HR (95% CI)	0.98 (0.96 to 1.00)	0.98 (0.95 to 1.01)	0.95 (0.89 to 1.02)	0.95 (0.91 to 0.98)	0.98 (0.93 to 1.04)	—
*P*_trend_	.02	.20	.16	.01	.53	.73
*P*_heterogeneity (compared to subtype 1)_^c^	—	—	.72	.39	.88	—

a Inverse probability weighting was applied to reduce a bias due to the availability of tumor tissue after cancer diagnosis (see Statistical Analysis subsection for details). Age- and cohort-stratified Cox proportional hazards model was used with further adjustment for race (White or nonWhite), height (continuous), family history of colorectal cancer (yes or no), history of lower gastrointestinal endoscopic exams (yes or no), body mass index (continuous), pack-years of smoking (continuous), physical activity (continuous), alcohol intake (continuous), and regular aspirin use (yes or no). BMI = body mass index; CI = confidence interval; CIMP = CpG island methylator phenotype; CRC = colorectal cancer; HPFS = Health Professionals Follow-up Study; HR = hazard ratio; NHS = Nurses’ Health Study; METS = metabolic equivalent task score; MIS = microsatellite instability.

b Subtype 1 (conventional pathway; non-MSI-high, CIMP-lowor negative, *BRAF*-wild-type, and *KRAS*-wild-type); Subtype 2 (serrated pathway; any MSI status, CIMP-high, *BRAF*-mutated, and *KRAS*-wild-type); Subtype 3 (alternate pathway; non-MSI-high, CIMP-lowor negative, *BRAF*-wild-type, and *KRAS*-mutated); Subtype 4 (other marker combinations).

c Heterogeneity across the 4 subtypes was tested by a weighted likelihood ratio test.

d For physical activity, the follow-up started in 1986 in NHS.

e BMI at age 18/21, BMI at age 18 for female and age 21 for male.

**Table 4.** Multivariable associations of dietary factors with risk of colorectal cancer according to molecular subtypes in the NHS and HPFS^a^

Risk factors	All CRC (n = 3063)	Subtype 1^b^ (Conventional pathway) (n = 498)	Subtype 2^b^ (Serrated pathway) (n = 138)	Subtype 3^b^ (Alternate pathway) (n = 367)	Subtype 4^b^ (Other pathway) (n = 172)	*P*_overall_ _heterogeneity_ ^c^
Total fiber intake, per 30 g/d						
HR (95% CI)	0.87 (0.67 to 1.14)	1.01 (0.48 to 2.13)	0.75 (0.23 to 2.43)	0.75 (0.36 to 1.56)	1.62 (0.54 to 4.87)	—
*P_t_*_rend_	.32	.99	.63	.44	.39	.68
*P*_heterogeneity (compared to subtype 1)_^c^	—	—	.67	.56	.47	—
Whole grain intake, per 20 g/d						
HR (95% CI)	0.92 (0.86 to 0.98)	0.77 (0.65 to 0.92)	0.93 (0.65 to 1.32)	1.15 (0.94 to 1.42)	0.92 (0.68 to 1.24)	—
*P*_trend_	.01	.003	.68	.18	.58	.03
*P*_heterogeneity (compared to subtype 1)_^c^	—	—	.36	.003	.32	—
Cereal fiber intake, per 5 g/d						
HR (95% CI)	0.92 (0.83 to 1.02)	0.76 (0.59 to 0.98)	1.26 (0.79 to 2.00)	1.08 (0.80 to 1.46)	1.02 (0.66 to 1.59)	—
*P*_trend_	.11	.03	.33	.61	.93	.15
*P*_heterogeneity (compared to subtype 1)_^c^	—	—	.06	.07	.26	—
Vitamin D intake, per 400 IU/d						
HR (95% CI)	0.81 (0.75 to 0.88)	0.70 (0.56 to 0.89)	0.69 (0.46 to 1.03)	0.76 (0.58 to 1.00)	1.00 (0.69 to 1.44)	—
*P*_trend_	<.001	.004	.07	.05	.98	.44
*P*_heterogeneity (compared to subtype 1)_^c^	—	—	.91	.66	.12	—
Marine n-3 fatty acid intake, per 0.2 g/d						
HR (95% CI)	0.99 (0.93 to 1.05)	1.00 (0.84 to 1.18)	0.73 (0.54 to 1.00)	0.84 (0.70 to 1.02)	0.99 (0.75 to 1.31)	—
*P*_trend_	.72	.95	.05	.08	.94	.26
*P*_heterogeneity (compared to subtype 1)_^c^	—	—	.09	.20	.98	—
Total red meat intake, per 3 serving/wk						
HR (95% CI)	1.05 (1.01 to 1.09)	1.19 (1.08 to 1.30)	0.99 (0.81 to 1.22)	0.96 (0.85 to 1.08)	1.06 (0.90 to 1.24)	—
*P*_trend_	.01	<.001	.94	.52	.51	.04
*P*_heterogeneity (compared to subtype 1)_^c^	—	—	.12	.006	.22	—
Processed red meat intake, per 3 serving/wk						
HR (95% CI)	1.14 (1.06 to 1.23)	1.45 (1.18 to 1.78)	0.96 (0.64 to 1.44)	0.88 (0.68 to 1.12)	1.11 (0.78 to 1.57)	—
*P*_trend_	<.001	<.001	0.85	0.30	0.58	.01
*P*_heterogeneity (compared to subtype 1)_^c^	—	—	.08	.002	.19	—
Unprocessed red meat intake, per 3 serving/wk						
HR (95% CI)	1.03 (0.97 to 1.09)	1.17 (1.00 to 1.36)	0.92 (0.68 to 1.25)	0.91 (0.76 to 1.10)	1.02 (0.78 to 1.33)	—
*P*_trend_	.34	.05	.61	.33	.88	.19
* P*_heterogeneity (compared to subtype 1)_ ^c^	—	—	.18	.04	.40	—
Total folate intake, per 400 µg/d						
HR (95% CI)	0.83 (0.76 to 0.90)	0.77 (0.59 to 1.00)	0.88 (0.58 to 1.33)	0.82 (0.63 to 1.07)	1.02 (0.72 to 1.43)	—
*P*_trend_	<.001	.05	.55	.14	.93	.63
*P*_heterogeneity (compared to subtype 1)_^c^	—	—	.58	.72	.20	—
Calcium intake, per 300 mg/d						
HR (95% CI)	0.91 (0.87 to 0.94)	0.81 (0.73 to 0.90)	0.92 (0.77 to 1.11)	0.89 (0.80 to 0.99)	1.02 (0.88 to 1.20)	—
*P*_trend_	<.001	<.001	.38	.04	.76	.11
*P*_heterogeneity (compared to subtype 1)_^c^	—	—	.25	.22	.02	—
Empirical dietary inflammatory pattern (EDIP) score, per 1 unit						
HR (95% CI)	1.28 (1.10 to 1.49)	1.25 (0.82 to 1.90)	1.37 (0.69 to 2.72)	0.53 (0.33 to 0.85)	1.18 (0.57 to 2.42)	—
*P*_trend_	.001	.31	.37	.01	.65	.03
*P*_heterogeneity (compared to subtype 1)_^c^	—	—	.82	.007	.90	—
Empirical dietary index for hyperinsulinemia (EDIH), per 1 unit						
HR (95% CI)	1.20 (1.01 to 1.41)	1.37 (0.88 to 2.15)	1.15 (0.50 to 2.64)	0.65 (0.40 to 1.07)	1.53 (0.74 to 3.19)	—
*P*_trend_	.03	.17	.74	.09	.25	.11
*P*_heterogeneity (compared to subtype 1)_^c^	—	—	.71	.03	.80	—

a Inverse probability weighting was applied to reduce a bias due to the availability of tumor tissue after cancer diagnosis (see Statistical Analysis subsection for details). Age- and cohort-stratified Cox proportional hazards model was used with further adjustment for race (White or nonWhite), height (continuous), family history of colorectal cancer (yes or no), history of lower gastrointestinal endoscopic exams (yes or no), body mass index (continuous), pack to years of smoking (continuous), physical activity (continuous), alcohol intake (continuous), and regular aspirin use (yes or no). For dietary factors, test for trend was conducted using the median of each quintile as a continuous variable. CI = confidence interval; CIMP = CpG island methylator phenotype; CRC = colorectal cancer; HPFS = Health Professionals Follow-up Study; HR = hazard ratio; MSI = microsatellite instability; NHS = Nurses’ Health Study.

b Subtype 1 (conventional pathway; non-MSI-high, CIMP-low/or negative, BRAF-wild-type, and KRAS-wild-type); Subtype 2 (serrated pathway; any MSI status, CIMP-high, BRAF-mutated, and KRAS-wild-type); Subtype 3 (alternate pathway; non-MSI-high, CIMP-low/or negative, BRAF-wild-type, and KRAS-mutated); Subtype 4 (other marker combinations).

c Heterogeneity across the 4 subtypes was tested by a weighted likelihood ratio test.

### Demographic and Clinical Factors

A statistically significant overall heterogeneity was found for age (*P*_overall heterogeneity_ = .001) and sex (*P*_overall heterogeneity_ < .001) (Table 2), with the highest hazard ratio observed for subtype 2 (HR per 10 years of age = 2.64, 95% CI = 2.13 to 3.26; HR for female sex = 2.65, 95% CI = 1.60 to 4.39) and the lowest hazard ratio for subtype 1 (HR = 1.70, 95% CI = 1.51 to 1.92 for age; HR = 0.74, 95% CI = 0.59 to 0.92 for female sex). The use of endoscopic exams was associated with lower risk of all subtypes except subtype 2. No difference by subtypes was found for the positive association with family history of CRC and inverse association with regular aspirin use.

### Anthropometric and Lifestyle Factors

Smoking was more strongly associated with higher risk of subtype 2 (HR per 20 pack-year = 1.29, 95% CI = 1.14 to 1.45; *P*_trend_ < .001) than other subtypes (*P*_overall heterogeneity_ < .001) (Table 3). For anthropometric measures, height was most strongly associated with subtype 2 (HR per 10 cm = 1.52, 95% CI = 1.21 to 1.90; *P*_trend_ < .001) than other subtypes; adulthood BMI and waist circumference were most strongly associated with subtype 1 in men and subtype 3 in women, although none of the heterogeneity tests was statistically significant. The multivariable hazard ratios per 5 kg/m^2^ increment in adulthood BMI were 1.42 (95% CI = 1.21 to 1.67) for subtype 1 in men and 1.23 (95% CI = 1.08 to 1.41) for subtype 3 in women. No evidence of subtype heterogeneity was found for alcohol or physical activity.

### Dietary Factors and Dietary Pattern

A stronger inverse association with subtype 1 than other subtypes was found for several individual dietary factors (Table 4), including whole grain intake (HR per 20 g/day, = 0.77, 95% CI = 0.65 to 0.92; *P*_trend_ = .003), cereal fiber intake (HR per 5 g/day = 0.76, 95% CI = 0.59 to 0.98; *P*_trend_ = .03), total vitamin D intake (HR per 400 IU/day = 0.70, 95% CI = 0.56 to 0.89; *P*_trend_ = .004), total folate intake (HR per 400 µg/day = 0.77, 95% CI = 0.59 to 1.00; *P*_trend_ = .04), and total calcium intake (HR per 300 mg/day = 0.81, 95% CI = 0.73 to 0.90; *P*_trend_ <.001). Similarly, a stronger positive association with subtype 1 was observed for total red meat (HR per 3 serving/week = 1.19, 95% CI = 1.08 to 1.30; *P*_trend_ < .001), and processed red meat (HR per 3 serving/week = 1.45, 95% CI = 1.18 to 1.78; *P*_trend_ < .001). In contrast, marine n-3 fatty acid intake and EDIP showed a stronger inverse and positive association, respectively, with subtype 2 CRC than other subtypes, although the heterogeneity test was not statistically significant.

## Discussion

In this study, we evaluated 24 risk factors in relation to risk of CRC according to pathway-specific molecular subtypes by leveraging the transdisciplinary molecular pathological epidemiology research approach, which enables investigators to link risk factors with tumor molecular signatures, refines effect size estimates, and enhances causal inference (23,24). We found that, although CRCs arising from different molecular pathways shared most of the risk factors, the magnitude of associations for some risk factors differed by tumor molecular subtypes. Our findings provide further evidence for the etiologic heterogeneity of CRC and have implications for better understanding the mechanisms through which risk factors influence CRC risk.

We found that age and female sex were more strongly associated with higher risk of subtype 2 than other subtypes, whereas male sex was strongly associated with higher risk of subtype 1 CRC. These findings are consistent with prior data that subtype 2 (serrated CRC) is more likely to occur in females and older individuals (25). Although the exact mechanisms remain unclear, the sex and age differences in epigenetic alterations, which are particularly important for the development of serrated CRC, have been proposed as a potential explanation (25).

In this study, we found that the inverse association of endoscopic exams with CRC varied by tumor subtypes, with the strongest association found for subtype 1 and no statistically significant association for subtype 2 (HR = 0.48 and 0.96, respectively). This is consistent with prior data indicating the limited protection of endoscopy against serrated polyps, which are more likely to be missed or incompletely removed endoscopically because of their predilection for proximal colon along with their flat and pale appearances (26). However, as with all observational studies, we cannot rule out unmeasured confounding related to bowel preparation, compliance with the screening guideline, and changes in screening practices. Moreover, we did not collect information on fecal occult blood test or fecal immunochemical test and therefore were unable to evaluate their associations with subtype-specific CRC. Further studies are needed to examine the influence of CRC screening on subtype-specific CRC risk.

Regular aspirin use has been established to reduce CRC incidence and mortality (27). In this study, we did not find substantial difference in the association of regular aspirin use with CRC risk according to tumor subtypes. Prior studies have found that the protective association of aspirin with CRC did not statistically significantly differ by *KRAS*, CIMP, or MSI status (28). Moreover, the beneficial association of aspirin with CRC has been primarily observed in tumors with high expression of prostaglandin-endoperoxide synthase 2 (also known as cyclooxygenase-2) (27), whose distribution does not appear to differ substantially across the 4 subtypes examined in this study (Supplementary Table 2, available online).

In the current study, we found that pack-years of smoking and pack-years of smoking before age 30 years were more strongly associated with higher risk of subtype 2 than other molecular subtypes. Prior studies have linked smoking to increased risk of CRC that harbors molecular features of the serrated pathway, such as CIMP, MSI, and *BRAF* mutation (6,29). Similarly, smoking has been more strongly associated with serrated polyps than with conventional adenomas (5,30). A possible explanation for this may relate to the activity of smoking in promotion of aberrant DNA promoter methylation that further leads to other molecular alterations commonly observed in subtype 2 CRC, such as MSI-high and CIMP-high (31,32).

Obesity is an established risk factor for CRC. In the current study, we found a stronger positive association for BMI and waist circumference with subtype 1 than other CRC subtypes in men. These findings are consistent with the accumulating evidence for a stronger association of BMI and waist circumference with non-MSI-high or *BRAF*-wild-type CRC (33,34). Several mechanisms have been proposed to explain the association between obesity and increased risk of CRC, including the insulin-like growth factor system, adipokines (eg, leptin, adiponectin), oxidative stress, and steroid hormones (35). Experimental evidence demonstrated that an obesity-causing mutation in the leptin receptor promoted the development of CRC in mice model with *APC* mutation, an important initiating event of subtype 1, whereas in MSI mice model, diet or weight change had no such effect (36,37).

Interestingly, in women, we found that BMI was strongly associated with higher risk of subtype 3. This finding is in line with a prior study that showed a stronger positive association of BMI with *KRAS*-mutated than *KRAS*-wild-type CRC in women (38). Inamura et al. reported that lower levels of adiponectin might underlie the effect of obesity on the development of *KRAS*-mutated CRC in women (39). However, given the limited data, more studies are needed to better understand the underlying mechanisms.

Several dietary factors have been implicated in the development of CRC (15). In the current study, we found that most dietary factors were more strongly associated with subtype 1 than other subtypes. This finding is consistent with our prior data that dietary factors were generally more strongly associated with conventional adenomas than serrated polyps (5). We speculate that the etiologic relevance of diet to CRC may differ by molecular subtypes. For example, we found that higher intake of red meat was associated with increased risk of subtype 1, in agreement with prior data that red meat consumption tends to be associated with *KRAS*-wild-type, *BRAF*-wild-type, CIMP-lowor negative, and non-MSI-high tumors (40,41). One possible explanation may be that red meat prepared at high temperatures is a major source of heterocyclic amines, a known mutagenic and carcinogenic agent. It has been shown that heterocyclic amines promote the development of chromosome instability (42), a key molecular alteration in subtype 1 (43).

EDIP is an index that characterizes the inflammatory potential of diet based on circulating inflammatory markers (44). In the current study, we found the positive association between EDIP and CRC appeared stronger for subtype 2 than for other subtypes. This finding is consistent with increasing data supporting that serrated pathway CRC may be more strongly associated with inflammatory diet, antitumor immune response, and alterations in the gut microbiome (45,46). Indeed, immunotherapy with monoclonal antibody to PDCD1 (programmed cell death 1, PD-1) has been approved for treatment of MSI-high CRC (47,48). In line with these data, we also found that higher intake of marine n-3 fatty acids, with potent anti-inflammatory properties, was associated with lower risk of subtype 2 but no other subtypes.

To the best of our knowledge, the current study represents the first effort to comprehensively characterize risk factor profiles of CRC according to major molecular subtypes. The strengths of our study include the prospective design, relatively large sample size, and repeated assessment of risk factors over 30 years. We used the IPW (19,20,49) method to reduce the potential bias by the availability of CRC tissue. The tumor molecular analyses were extensively validated with their performance characteristics documented (12–14). Our molecular pathological epidemiology approach could uncover possible novel links between risk factors and molecular subtypes and provide pathogenic insights (23,24).

Our study also has some limitations. First, multiple comparisons were performed, and thus some of the findings may be due to chance. However, we adopted the recently proposed stringent α level of 0.005 (22). Also, all of the risk factors and statistical comparisons were set a priori based on previous data. We also interpret our results in a holistic manner, considering biological plausibility, coherence, and consistency rather than statistical significance in isolation. Second, lifestyle and dietary factors were all self-reported and thus subject to measurement error. However, given the prospective design, any error in exposure assessment would have likely attenuated the observed associations toward the null hypotheses. Third, all of the study participants were health professionals and largely White, which may limit the generalizability of our findings. However, most of our previously reported risk factor associations with CRC have been replicated by other cohorts (50). Finally, because of the exploratory nature of our study, some risk factors did not achieve statistical significance, and thus the discussion about these factors is speculative and needs to be confirmed in further studies.

In this study of 3063 patients of CRC, we found that risk factor profiles may be different in the 4 molecular subtypes. The finding, although requiring replication in independent studies, suggests the distinct role of some risk factors in CRC arise through different pathways and have implications for better understanding the etiology of CRC and improving precise and effective prevention strategies.

## Funding

This work was supported by the American Cancer Society Mentored Research Scholar Grant (MRSG-17-220-01- NEC to MS); the US National Institutes of Health (P01 CA87969 to MJ Stampfer; UM1 CA186107 to MJ Stampfer; P01 CA55075 to WC Willett; UM1 CA167552 to WC Willett; U01 CA167552 to WC Willett and LA Mucci; K07 CA188126 to XZ; P50 CA127003 to CS Fuchs; K24 DK098311, R01 CA137178, R01 CA202704, R01 CA176726 to ATC; R01 CA151993, R35 CA197735 to SO; R03 CA197879 to KW; R21 CA230873 to KW and SO; R00 CA215314 to MS); and the American Cancer Society Research Scholar Grant (RSG NEC-130476 to XZ), American Institute for Cancer Research Grant to KW, the Project P Fund for Colorectal Cancer Research, The Friends of the Dana-Farber Cancer Institute, Bennett Family Fund, and the Entertainment Industry Foundation through National Colorectal Cancer Research Alliance. ATC is a Stuart and Suzanne Steele MGH Research Scholar. JAM research is supported by the Douglas Gray Woodruff Chair fund, the Guo Shu Shi Fund, Anonymous Family Fund for Innovations in Colorectal Cancer, and the George Stone Family Foundation. TU was supported by a grant from Overseas Research Fellowship (201960541) from Japan Society for the Promotion of Science. TU, KH, KF was supported by fellowship grants from the Uehara Memorial Foundation. KH was supported by the Mitsukoshi Health and Welfare Foundation. KF was supported by Grant of the Clinical Research Promotion Foundation (2018). LW was supported by the “3&3 Team Project” of the First Affiliated Hospital of Sun Yat-sen University.

## Notes

**Role of the funders:** The funders had no role in design and conduct of the study; collection, management, analysis, and interpretation of the data; and preparation, review, or approval of the manuscript. The content is solely the responsibility of the authors and does not necessarily represent the official views of the National Institutes of Health.

**Disclosures:** Andrew T. Chan previously served as a consultant for Bayer Pharma AG, Pfizer In., and Janssen Pharmaceuticals for work unrelated to the topic of this manuscript. This study was not funded by Bayer Pharma AG, Pfizer Inc, or Janssen Pharmaceuticals. Jeffrey A. Meyerhardt has received institutional research funding from Boston Biomedical, has served as an advisor/consultant to Ignyta and COTA Healthcare, and served on a grant review panel for the National Comprehensive Cancer Network funded by Taiho Pharmaceutical. No other conflict of interest exists.

**Acknowledgments:** We would like to thank the following state cancer registries for their help: AL, AZ, AR, CA, CO, CT, DE, FL, GA, ID, IL, IN, IA, KY, LA, ME, MD, MA, MI, NE, NH, NJ, NY, NC, ND, OH, OK, OR, PA, RI, SC, TN, TX, VA, WA, WY. The authors assume full responsibility for analyses and interpretation of these data.

**Author contributions:** Study concept and design: LW, XH, SO, MS; acquisition of data: XH, CHL, DH, KW, MW, ATC, ELG, SO, MS; statistical analysis: LW; interpretation of data: all authors; drafting of the manuscript: LW, MS; critical revision of the manuscript for important intellectual content: XH, TU, KH, CHL, DH, NA, KF, CSF, JAM, XZ, KW, ATC, ELG, SO.

## Data Availability

The data that support the findings of this study are available on request at https://www.nurseshealthstudy.org/researchers (email: nhsaccess@channing.harvard.edu) and https://sites.sph.harvard.edu/hpfs/for-collaborators/. The data are not publicly available due to privacy or ethical restrictions.

## Supplementary Material

pkaa089_Supplementary_Data
