# Supplementary material for: Risk Factors and Incidence of Colorectal Cancer According to Major Molecular Subtypes
Source: JNCI Cancer Spectr. 2020 Oct 7;5(1):pkaa089. doi: 10.1093/jncics/pkaa089 (PMC7791624; doi:10.1093/jncics/pkaa089)
Supplement: pkaa089_Supplementary_Data [file pkaa089_supplementary_data.pdf]

Supplementary Materials

Supplementary Methods

Assessment of risk factors

For smoking, we assessed pack-years of smoking up to date and pack-years smoked before age 30 to account for the life-course effect. In addition to current body weight assessed on the biennial questionnaires, we asked participants to recall their body weight at age 18 in 1980 in the NHS, and at age 21 in 1986 in the HPFS. We used these data along with height reported at baseline to calculate BMI at young adulthood. In the NHS, women were asked to report their waist circumferences using a tape measure in 1986, 1996 and 2000. In the HPFS, we enclosed a tape measure in an optional questionnaire and directed participants to measure their waist at the umbilicus in 1987 and 1996. We assessed every 4 years the type and duration spent on specific physical activities and calculated total physical activity as the summed metabolic equivalent of task (MET) hours per week.

Assessment of Family history of CRC

We utilized family history data prospectively collected from questionnaires before a participant developed colorectal cancer, to avoid recall bias. If a participant did not develop colorectal cancer, we utilized questionnaire data throughout the follow-up period or until death. In our cohort studies, we classified participants as having a family history of CRC if they reported at least 1 first-degree relative (parent, sibling, or child) with CRC.

Assessment of the use of endoscopic exams

In both cohorts, beginning in 1988 and continuing through 2002, participants were asked biennially whether they had undergone lower gastrointestinal endoscopy and, if so, the reason for the endoscopy. In 2004, we additionally inquired whether the previously reported endoscopies were sigmoidoscopies or colonoscopies. Every cycle thereafter, responses for sigmoidoscopy and colonoscopy were recorded separately. For our study, we considered endoscopic use for the screening purpose only.

Molecular pathological epidemiology (MPE) database and inverse probability weighting (IPW)

Molecular pathological epidemiology (MPE) database of colorectal cancer are based on two U.S. nationwide prospective cohort studies (NHS, HPFS) and integrates various data about colorectal cancer patients, including the clinicopathological and molecular features, long-term survival data, and lifestyle information. This comprehensive database allowed us to examine an interactive relationship between a specific environmental exposure and CRC subtypes while at the same time adjusting for a variety of potential confounders. This MPE database can provide etiologic and pathogenic insights, potentially contributing to precision medicine for personalized prevention and treatment.

To account for the missing data on tumor molecular markers, we performed the IPW analysis in the Cox proportional hazards regression. The method details have been described in our previous publication.[3] Briefly, we first modeled the data availability of molecular subtypes (based on a combinatorial status of microsatellite instability (MSI), CpG island methylator phenotype (CIMP), and *BRAF* and *KRAS* mutations) using logistic regression with the subtype availability

status (subtype data available vs. missing) as the outcome variable, and covariates that may influence the success of tissue collection as predictor variables, including disease stage (stage I, stage II, stage III, or stage IV), tumor location (proximal colon, distal colon, or rectum), tumor differentiation (well differentiation, moderated differentiation, or poor differentiation), age at diagnosis (continuous), age at diagnosis-square (continuous), diagnostic year (continuous), and family history of colorectal cancer (yes or no). We then calculated the weight as the inverse of the probability of molecular subtype data availability estimated from the logistic model. Extremely small and large weights were truncated at the 5th/95th percentile to reduce outlier effects. We then incorporated this inverse probability weight into our Cox proportional hazards regression model. Weight was integrated in the variance estimation using the robust sandwich variance estimate.[4]

Statistical analysis

We excluded participants who had missing data on any of the studied risk factors at baseline. For missing data that occurred in the follow-up questionnaires, we carried forward the most recent available information from prior questionnaires. To reduce intra-individual variation and capture long-term exposures, we used the cumulative average for relevant variables, which was the mean of all available data prior to each of the questionnaire cycles. To control for confounding by age, calendar time, and any possible two-way interactions between these two-time scales, we stratified the analysis jointly by age and calendar time of the current questionnaire cycle. Proportional hazards assumption was tested by including the product term between exposure variable and time in the model and testing the significance of the product term using the Wald test. No deviation from the proportional hazards assumption was detected. All models were

adjusted for race (white or non-white), height (continuous), family history of colorectal cancer (yes or no), history of endoscopic exams (yes or no), body mass index (continuous), pack-years of smoking (continuous), physical activity (continuous), alcohol intake (continuous), and regular aspirin use (yes or no).

The basic model for our analysis was the proportional hazards model, $I(t\,|x,U,i)=I_{0i}(t)\exp\{\beta_1 x(t)+\beta_2 U(t)\}$, where β_1 is the $\log_e$ incidence rate ratio describing the increase or decrease in the baseline incidence rate at time t due to a one unit increase in exposure $x(t)$ measured at time t, $U(t)$ is a vector of covariates at age t, β_2 is the vector of $\log_e$ incidence rate ratio describing the increase or decrease in the baseline incidence rate due to a one unit increase in these *a priori* covariates, t is the age at which the cancer of interest is diagnosed, and $I_{0i}(t)$ is the baseline cancer incidence rate at age t in stratum i. Person-years of follow-up was counted from the age in months at the date the baseline questionnaire was returned until the age in months at the date of diagnosis of CRC, age at date of death or age at end of follow-up, whichever came first. SAS PROC PHREG was used for all analysis and the Anderson-Gill data structure was used to handle time-varying covariates efficiently, where a new data record is created for every questionnaire cycle at which a participant was at risk, with covariates set to their values at the time that the questionnaire was returned. To control as finely as possible for confounding by age, calendar time and any possible two-way interactions between these two-time scales, we stratified the analysis jointly by age in months at start of follow-up and calendar year of the current questionnaire cycle. The time scale for the analysis was then measured as months since the start of the current questionnaire cycle, which is equivalent to age in months because of the way we structured the data and formulated the model for analysis.

Reference

1. Giovannucci E, Ascherio A, Rimm EB, Colditz GA, Stampfer MJ, Willett WC. Physical activity, obesity, and risk for colon cancer and adenoma in men. Ann Intern Med. 1995;122(5):327-334.
2. Wark PA, Wu K, van 't Veer P, Fuchs CF, Giovannucci EL. Family history of colorectal cancer: a determinant of advanced adenoma stage or adenoma multiplicity? Int J Cancer. 2009;125(2):413-420.
3. Liu L, Nevo D, Nishihara R, et al. Utility of inverse probability weighting in molecular pathological epidemiology. European journal of epidemiology 2018;33(4):381-92. doi: 10.1007/s10654-017-0346-8
4. Lin DY, Wei LJ (1989) The Robust Inference for the Proportional Hazards Model. Journal of the American Statistical Association 84: 1074–1078 doi:10.2307/2290085.

Supplementary table 1 Basic characteristics of colorectal cancer patients with and without tumor molecular information in the NHS and HPFS [a]

Risk factors	CRC with molecular subtype information	CRC without molecular subtype information
	n=1175	n=1888
Age, years	67.2 (8.9)	67.6 (10.2)
Whites, %	94	95
Female, %	60	57
Family history of CRC, %	26	23
Regular aspirin use, % [b]	40	38
Height, cm	170 (9)	170 (9)
Body mass index, kg/m2	26.4 (4.5)	26.5 (5.0)
BMI at age 18/21, kg/m2	22.0 (3.8)	22.0 (3.9)
Waist circumference, cm	90.5 (13.5)	91.3 (14.0)
Pack-years of smoking	16. 9 (21.6)	16.9 (22.6)
Pack-years of smoking before age 30	4.9 (6.2)	5.0 (6.4)
Physical activity, MET-hours/week [c]	19.1 (16.1)	19.3 (16.2)
Alcohol intake, g/day	9.7 (13.9)	9.2 (12.6)
Total folate intake, µg/day	462 (203)	469 (223)
Calcium intake, mg/day	921 (357)	921 (365)
Vitamin D intake, IU/day	376 (234)	379 (225)
Marine omega-3 fatty acid intake, g/day	0.24 (0.21)	0.25 (0.21)
Total red meat intake, serving/week	6.3 (3.6)	6.5 (4.2)
Processed red meat intake, serving/week	2.2 (2.0)	2.3 (2.4)
Unprocessed red meat intake, serving/week	3.9 (2.3)	4.0 (2.5)
Total fiber intake, g/day	19.2 (6.1)	19.1 (6.2)
Whole grain intake g/day	20.5 (14.2)	21.4 (15.4)
Cereal fiber intake, g/day	5.16 (2.65)	5.07 (2.71)
Empirical dietary inflammatory pattern score	-0.01 (0.33)	0.01 (0.34)
Empirical dietary index for hyperinsulinemia	0.35 (0.25)	0.37 (0.26)

Abbreviations: CRC, colorectal cancer; HPFS, the Health Professionals Follow-up Study; MET, metabolic equivalent task; NHS, the Nurses' Health Study.

[a] Data are information for each CRC at diagnosis. All variables are adjusted for age and sex except for age and sex themselves. Mean (standard deviation) is presented for continuous.

[b] A standard tablet contains 325 mg aspirin, and regular users were defined as those who used at least two standard tablets per week.

[c] Physical activity is calculated by the product sum of the MET of each specific recreational activity and hours spent on that activity per week. For physical activity, the follow-up started in 1986 in NHS.

Supplementary Table 2 Distribution of prostaglandin-endoperoxide synthase 2 (PTGS2) expression according to molecular subtypes in the NHS and HPFS

Risk factors	Participants with CRC				
	All CRC	Subtype 1 (Conventional pathway)	Subtype 2 (Serrated pathway)	Subtype 3 (Alternate pathway)	Subtype 4 (Other pathway)
PTGS2	n=1175	n=498	n=138	n=367	n=172
Negative, n (%)	389 (33)	137 (28)	62 (45)	114 (31)	76 (44)
Positive, n (%)	651 (55)	313 (62)	51 (37)	206 (56)	81 (47)
Missing, n (%)	135 (12)	48 (10)	25 (18)	47 (13)	15 (9)

Abbreviations: PTGS2, prostaglandin-endoperoxide synthase 2; HPFS, the Health Professionals Follow-up Study; NHS, the Nurses' Health Study.

Subtype 1 (conventional pathway; non-MSI-high, CIMP-low/negative, BRAF-wildtype, and KRAS-wildtype),

Subtype 2 (serrated pathway; any MSI status, CIMP-high, BRAF-mutated, and KRAS-wildtype),

Subtype 3 (alternate pathway; non-MSI-high, CIMP-low/negative, BRAF-wildtype, and KRAS-mutated),

Subtype 4 (other marker combinations).
